# MHD micro polar fluid flow over a stretching surface with melting and slip effect

**DOI:** 10.1038/s41598-023-36988-3

**Published:** 2023-07-03

**Authors:** Surbhi Sharma, Amit Dadheech, Amit Parmar, Jyoti Arora, Qasem Al-Mdallal, S. Saranya

**Affiliations:** 1 Department of Mathematics, Swami Keshvanand Institute of Technology, Management and Gramothan, Jaipur, India; 2 Department of Mathematics, Arya College of Engineering and IT, Jaipur, India; 3 Department of Mathematical Sciences, UAE University, P.O. Box 15551, Al Ain, Abu Dhabi, UAE

**Keywords:** Materials science, Mathematics and computing

## Abstract

Objective of the present analysis is to represent the phenomenon of Heat–mass transfer on MHD micro polar fluids caused by permeable and continuously stretching sheet along with slip impacts fostered in a porous medium. Consequently, the equation of energy includes the term of non-uniform heat source/sink. The equation regarding species concentration in cooperates the terms indicating order of chemical reaction to characterize the chemically reactive species. The application software MATLAB with governing syntax of bvp4c technique are employed to reduce equations of momentum, micro-rations, heat, and concentration into suitable required simplifications to derive necessary arithmetic manipulations of available non-linear equations. Various dimensionless parameters are portrayed in the available graphs with essential consequences. Analysis discovered that micro-polar fluid improves velocity and temperature profile while it suppresses micro-rations profile also magnetic parameter ($M$) and porosity parameter ($K_p$) reduces the momentum boundary layer thickness. The acquired deductions verify remarkable correspondence with already reported in an open literature.

## Introduction

In recent past academic attainment of micro-polar fluid has drawn attracted attention among several engineering community and scientist community as a reason of its limited circumference associated with Newtonian fluids. These fluids are influentially determined by spin inertia and reinforces stress moments and body moments. The theory of microfluids is identified as complex theory against the case of constitutively linear theory and the corresponding underlying mathematical manipulations are not easily amenable to the solution of non-trivial problems in this field. A subclass of these fluids is defined as the micropolar fluids that exhibits micro-rotational effects and micro-rotational inertia. The classical framework of Navier–Stokes model founds certain degree of limitation particularly listing as it cannot describes and elaborates the category of fluids pertaining microstructure characteristics, fluids possessing effective and influential applications. Therefore, analysis of micro polar fluids suggested by Eringen^1^ offers definite model for fluids which possesses polymeric and rotating particles by comprehending micro rotational momentum equation together with classical momentum equation. Investigations of micro polar fluids are of significant recognition because of numerous applications in various industries particularly suspension solutions, solidification of liquid crystals, animal bloods, and exotic lubricants. Bhargava and Takhar^2^ explored heat transfer of the micro-polar boundary layer (BL) near a stagnation point on a moving wall. Anika et al.^3^ analyzed consequences of thermal diffusion on the unsteady viscous MHD micro-polar fluid flow past an infinite plate together with hall and ion-slip current. Bhargava et al.^4^ performed numerical investigations for micro-polar transfer phenomena prompted by non-linear stretching sheet availing two distinct techniques of finite element and finite difference. Takhar et al.^5^ exercised mixed convection in MHD flow of micro-polar fluids across the stretchy sheet. Bhargava and Rana et al.^6^ examined nonlinear convective heat and mass transfer in a micro-polar fluid with continuously variable conductivity by employing the objectives of finite element technique.

The flow of fluid across continuously stretching sheet under the influence of available magnetic field has significant emphasis on several domains of engineering particularly plasma investigations, geothermal energy extraction etc. Investigations pertaining to MHD effects on flow of fluid under consideration past a stretching sheet are indexed in an open literature. The first study by Crane^7^ has fascinated many researchers to investigate alike problems on the boundary layer (B.L.) flow due to a stretching sheet, as it has numerous applications in industry like the extrusion of polymer sheet by a dye, crystal growing, continuous casting and drawing of plastic films. The pace of cooling and the stretching process are the only factors that directly affect the desired properties of the finished product. The stretching sheet may not be necessarily linear, as we can take in nonlinear fashion also, even though problem may not have noticeable technological relevance. In view of this, Vajravelu^8^ proposed the flow across a nonlinearly stretching sheet, while Cortell^9,10^ studied the flow and heat**-**transport caused by a stretching sheet for two unalike types of thermal boundary (TB) conditions on the sheet, viz., constant surface temperature (CST) and prescribed surface temperature (PST). Ganji et al.^11^ reported analytical solution for magneto hydrodynamic flow due to a stretching sheet in nonlinearly manner. Similar work has been studied by Ishak et al.^12^, Prasad et al.^13^, Van Gorder et al.^14^, Raftari et al.^15^, Abbas and Hayat^16^, Dadheech et al.^17^, Olkha et al.^18^ and Abel et al.^19^, among others.

The consolidated impacts of heat mass diffusion together with chemical reaction has their dominant significance on several processes emerging in cooling of nuclear reactors, thermal insulation, geothermal reservoirs etc. Andersson et al.^20^ examined chemically reactive species diffusion due to a plane elastic surface. Abo-Eldahab and Salem^21^ studied flow and heat transfer of non-Newtonian power law fluid flow with mass diffusion and chemical reaction on a moving cylinder under consideration of magnetic field effect**.** Chauhan and Jakhar^22^ reported 2D non-Newtonian flow and heat transport in a channel with suction at the top and a naturally permeable medium at the bottom. Chauhan and Ghiya^23^ suggested heat-transfer in second order fluid flow in between two stable permeable disks together with the consequences of magnetic field. Kumar^24^ investigated analysis of finite element combined with heat-mass transfer in hydromagnetic micro-polar flow past a stretching sheet. Emad et al.^25^ explored the investigations of flowing/suction impacts on the hydromagnetic heat-transfer by the application of mixed convection from continuously stretching surface together with internal heat generation/absorption. Tripathy et al.^26^ examined the numerical evaluations of hydromagnetic micropolar fluids past the stretching sheet embedded in a porous channel together with non-uniform heat sources and permissible chemical reactions. Chen and Taiwan^27^ inspected the theory of heat-mass transfer in MHD flow prompted by natural convection from permeable and suitably inclined stretching surface embedded with variable temperature of wall and concentration. Alam et al.^28^ examined numerical proposals of combined free-forced convection and mass transfer flow past the available vertical, porous plate in the porous channel together with heat generation and thermal diffusions. Aydin and Kaya^29^ investigated the MHD mixed convective heat transfer flow about the suitably inclined plate. Reddy and Reddy^30^ suggested investigations of mass transfer and heat generation consequences on MHD free convection flow across the inclined vertical surface in porous medium. Patil et al.^31^ proposed the influential consequences of Eyring–Powell fluid across the stretching surface in the existence of magnetic field and chemical reactions.

Fundamental phenomenon of melting heat transfer finds dominant significance in various technological and industrial exercises like comprehending melting of permafrost, magma solidification, metal purification, welding etc. Epstein and cho et al.^32^ established melting impacts on the mechanism of heat transfer. Yacob et al.^33^ examined melting heat transfer in boundary layer stagnation point flow towards a stretching/shrinking sheet in a micropolar fluid. Hayat et al.^34^ examined Powell-Eyring stagnation point flow towards a surface stretching linearly with melting heat transfer. Melting heat and mass transport effects in non-Newtonian flow over a stretching surface with non-linear radiation and magnetic field effect was discussed by Khan et al.^35^. Gireesha et al.^36^ investigated melting heat transfer in MHD flow of dusty Casson fluid over a stretching surface.

A fluid sometimes gets adhered to the solid boundary but in some circumstances, it does not get a hold like as in suspensions, melting of polymers, emulsion processes and several other non-Newtonian fluids often exhibits macroscopic wall slip. Fluids which manifest boundary slip finds applications in various domains such as polishing of heart valves, internal cavities and various other technological procedures. Ali et al.^37^ investigated slip effects in viscoelastic fluid flow through porous medium due to a porous oscillatory stretched sheet. Govindarajan et al.^38^ discussed slip and mass transfer effects in a vertical channel under consideration of heat source and radiation. Olkha and Dadheech^39,40^ discussed entropy analysis for MHD flow for different non-Newtonian fluid caused by a stretching sheet with slip effect and heat source. Dadheech et al.^41^ investigated MHD flow for Casson fluid caused by a stretching sheet with slip effect. Dadheech et al.^42^ discussed entropy analysis for Williamson fluid caused by a vertical plate with Cattaneo-Christov heat flux and slip effect. The boundary layer flow for different fluids and geometrical configurations has been considered by^43–59^ in the presence of magnetic field.

In perspective of given literature review we have observed that there are relatively few studies are performed on MHD Micro-Polar fluid prompted by melting stretching sheet. The main objective of current study is to determine flow behavior and heat transfer of Micro-Polar over a melting stretching sheet. The novelty of the presented work is increased by substantial validating slip effects with chemical reaction and non-uniform heat source/sink. The examinations furnished in the given article can be further utilized to make investigations in fuel industries, flow of crushed water problems, and in the extrusion of polymer sheets. The consequences of the investigations made are employed in various engineering designs, metallurgy industries also for improving the working efficiency of systems for flow of thermos fluids.

### Mathematical formulation

**Figure 1:**
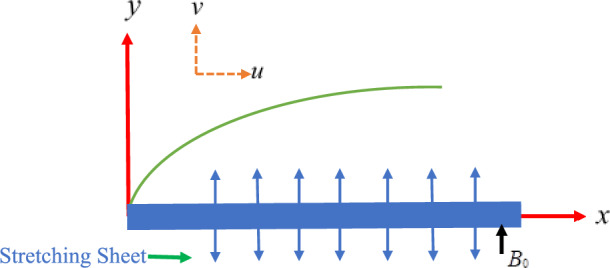
Physical model of the problem.

Steady two-dimensional incompressible micro-polar fluid flows caused by a stretching sheet are examined. Corresponding velocity components $u$ and $v$ along $x$ axis and $y$ axis and $N$ is corresponding component of micro-rotation as shown in Fig. 1. For micro-polar fluid governing system of equations administered by following Tripathy et al.^26^ with relevant boundary conditions are given as:

- In the momentum equation we take micropolar fluid, magnetic field and porous medium term. The magnetic field Bo is applied perpendicular to the stretching sheet and the effect of induced magnetic field is neglected since the magnetic Reynolds number is assumed to be small. We further assume that the impressed electric field is zero and Hall effect is neglected.
- The thermal contribution of non-uniform heat source and sink is introduced effectively in the energy equation.
- The mass transfer phenomenon due to diffusion of chemically reactive foreign species has been accounts for by considering the chemical reaction term of first order.
  1 $$\frac{\partial u}{{\partial x}} + \frac{\partial u}{{\partial y}} = 0$$

Continuity equation

2 $$u\frac{\partial u}{{\partial x}} + v\frac{\partial u}{{\partial y}} = \left( {\upsilon + \frac{{k_{v} }}{\rho }} \right)\frac{{\partial^{2} u}}{{\partial y^{2} }} + \frac{{k_{v} }}{\rho }\frac{\partial N}{{\partial y}} - \frac{{\sigma B_{0}^{2} }}{\rho }u - \frac{\upsilon }{{k_{p} }}u$$

Momentum equation

3 $$u\frac{\partial N}{{\partial x}} + v\frac{\partial N}{{\partial y}} = \frac{\gamma }{j\rho }\frac{{\partial^{2} N}}{{\partial y^{2} }} - \frac{{k_{v} }}{j\rho }\left( {2N + \frac{\partial N}{{\partial y}}} \right)$$

Angular momentum equation

4 $$u\frac{\partial T}{{\partial x}} + v\frac{\partial T}{{\partial y}} = \frac{{k_{f} }}{{\rho C_{p} }}\frac{{\partial^{2} T}}{{\partial y^{2} }} + \left( {\frac{{\mu + k_{v} }}{{\rho C_{p} }}} \right)\left( {\frac{\partial u}{{\partial y}}} \right)^{2} + \frac{{\sigma B_{0}^{2} }}{{\rho C_{p} }}u^{2} + \frac{{q^{\prime \prime \prime } }}{{\rho C_{p} }}$$

Energy equation

5 $$u\frac{\partial C}{{\partial x}} + v\frac{\partial C}{{\partial y}} = D\frac{{\partial^{2} C}}{{\partial y^{2} }} - k_{n} \left( {C - C_{\infty } } \right)$$

Species equationhere $u$ , $v$ are representing the component of velocity corresponding to the horizontal and the vertical direction respectively. $\rho$, $\upsilon$, $k_{f}$ $B_{0}$, $\sigma$, $k_{p}$, $T$, $C_{p}$, $C$, $D$, $k_{n}$ are listed as the density of fluid, kinematic viscosity, thermal conductivity, strength of magnetic field, electrical conductivity, permeability of a porous medium, temperature of fluid, specific heat, fluid’s concentration, coefficient of mass diffusion and parameter associated to chemical reaction respectively.

The appropriate boundary condition (Olkha et al.^39^) for flow, concentration and temperature is

6 $$\begin{aligned} & at\,y = 0\,\,\,\,\,\,\left\{ {\begin{array}{*{20}l} {u = u_{w} + L_{1} \frac{\partial u}{{\partial y}},\,\,v = \kappa \frac{1}{{\rho \left\{ {\beta_{m} + c_{s} (T_{m} - T_{0} )} \right\}}}\frac{\partial T}{{\partial y}} - v_{w} ,} \hfill \\ {N = - s\frac{\partial u}{{\partial y}}} \hfill \\ {T = T_{m} + L_{2} \frac{\partial T}{{\partial y}},\,\,\,\,\,} \hfill \\ {\,C = C_{w} + \,L_{3} \frac{\partial C}{{\partial y}}} \hfill \\ \end{array} } \right. \\ & a\,t\,\,y \to \infty \,\,\left\{ {\begin{array}{*{20}l} {u \to 0,\,\,\,\,\,\,\,N \to 0\,,\,\,} \hfill \\ {C \to C_{\infty } ,\,T \to T_{\infty } } \hfill \\ \end{array} } \right. \\ \end{aligned}$$

where $u_{w} ,\,N,\,\,s,\,\,L_{1} ,\,\,L_{2} ,\,\,L_{3} ,\,\,k_{v} ,\,\,\beta_{m} ,\,\,c_{s} ,\,\,T_{m} ,\,\,T_{0} ,\,\,C_{w} ,\,\,T_{\infty } \,,\,k_{p}$ and $v_{w} > 0$ are surface velocity, microrotation velocity, surface condition parameter, velocity slip, thermal slip and concentration slip parameter, microrotation viscosity, latent heat, heat capacity of solid surface, melting temperature, solid surface temperature, fluid concentration at the wall, free stream temperature, and suction velocity respetively. It is assumed that $\gamma = \left( {\mu + \frac{{k_{v} }}{2}} \right)j$ where $j = \frac{\nu }{b}$ as a reference length. The non-uniform heat source/Sink is considered following (Abo-Eldahab et al.^21^)

7 $$q^{\prime \prime \prime } = \frac{{\rho ku_{w} (x)}}{xK}\left[ {A^{*} (T_{w} - T_{\infty } )f^{\prime } + B^{*} (T - T_{\infty } )} \right]$$

Here,$A^{*} ,\,\,B^{*} > 0$ corresponds to internal heat generation, while, $A^{*} ,\,\,B^{*} < 0$ corresponds to internal heat absorption.

### Solution

Here we consider the similarity transformation relations of the following form:

8 $$\begin{gathered} \eta = \sqrt {\frac{b}{\upsilon }} y,\,\,\,\,\,\,\,\,\,\,\,\,\,\,\,\,\,\,\,\,\,\,\,\,u = bxf^{\prime } (\eta )\,,\,\,\,\,\,\,\,\,\,\,\,\,\,\,\,\,\,v = - \sqrt {b\upsilon } f(\eta ) \hfill \\ N = b\sqrt {\frac{b}{\upsilon }} xg(\eta )\,,\,\,\,\,\,\,\,\,\theta (\eta ) = \frac{{T - T_{\infty } }}{{T_{m} - T_{\infty } }}\,,\,\,\,\,\,\,\,\,\,\,C(\eta ) = \frac{{C - C_{\infty } }}{{C_{w} - C_{\infty } }}\, \hfill \\ \end{gathered}$$

The equation of continuity is satisfied, identically. Substitution of (8) in (2–5) results in the following non-linear ODE’s:

9 $$(1 + K)f^{\prime \prime \prime } + f\,f^{\prime \prime } - f^{{\prime}{2}} + K\,g^{\prime } - \left( {M + Kp} \right)f^{\prime } = 0$$

10 $$\left( {1 + \frac{K}{2}} \right)g^{\prime \prime } + fg^{\prime } - gf^{\prime } - K(2g + f^{\prime \prime } ) = 0$$

11 $$\theta^{\prime \prime } + \Pr \left( {f\theta^{\prime } - f^{\prime } \theta } \right) + (1 + K)\Pr \,Ec\,f^{\prime \prime 2} + \Pr \,Ec\,M\,f^{\prime 2} + A^{*} f^{\prime } + B^{*} \theta = 0$$

12 $$\phi^{\prime \prime } + Sc\,f\phi^{\prime } - Sc\,K_{n} \,\phi = 0$$

and the B.C. (6) are reduced as:

13 $$\begin{aligned} & at\,\,\eta = 0\,\,\,\,\,\left\{ {\begin{array}{*{20}l} {f(\eta ) = S - \frac{Me}{{\Pr }}\theta^{\prime } ,} \hfill \\ {f^{\prime } (\eta ) = 1 + \delta_{1} f^{\prime \prime } (\eta ),} \hfill \\ {g(\eta ) = - sf^{\prime \prime } (\eta )} \hfill \\ {\theta (\eta ) = 1 + \delta_{2} \theta^{\prime } (\eta ),\,\,\,\,} \hfill \\ {\,\phi (\eta ) = 1 + \delta_{3} \phi^{\prime } (\eta ),} \hfill \\ \end{array} } \right. \\ & as\,\,\eta \to \infty \,\,\,\left\{ {\begin{array}{*{20}l} {f^{\prime } (\eta ) \to 0,\,\,\theta (\eta ) \to 0,} \hfill \\ {\phi (\eta ) \to 0,\,\,\,\,g(\eta ) \to 0} \hfill \\ \end{array} } \right. \\ \end{aligned}$$

where Material (micropolar) fluid parameter $K = \frac{{k_{v} }}{\mu }$; Magnetic field parameter $M = \frac{{\sigma B_{0}^{2} }}{\rho b}$; Prandtl number $\Pr = \frac{{\rho \upsilon C_{p} }}{{k_{f} }}$; Eckert number $\,Ec = \frac{{u_{w}^{2} }}{{C_{p} (T_{w} - T_{\infty } )}}$; Schmidt number $\,Sc = \frac{\upsilon }{D}$, suction/injection coefficient $S = \frac{{V_{0} }}{{\sqrt {b\upsilon } }}\,$,$\,Kp = \frac{\upsilon }{{ak_{p} }}$, Porosity parameter, source dependent and temperature dependent parameter $A^{*}$ and $B^{*}$, Chemical reaction parameter $K_{n}$, velocity slip parameter $\delta_{1} = L_{1} \sqrt {{b \mathord{\left/ {\vphantom {b \upsilon }} \right. \kern-0pt} \upsilon }}$, temperature slip parameter $\delta_{2} = L_{2} \sqrt {{b \mathord{\left/ {\vphantom {b \upsilon }} \right. \kern-0pt} \upsilon }}$, mass slip parameter $\delta_{3} = L_{3} \sqrt {{b \mathord{\left/ {\vphantom {b \upsilon }} \right. \kern-0pt} \upsilon }}$, and melting surface parameter $Me = \frac{{\left( {T_{m} - T_{\infty } } \right)C_{p} }}{{\beta_{m} + c_{s} \left( {T_{m} - T_{0} } \right)}}$.

### Physical quantities of interest

The local “skin friction coefficient” $C_{f}$ defined as

14 $$C_{f} = \frac{{\tau_{w} }}{{(\rho u_{w}^{2} )}} = \frac{{(1 + K)f^{\prime \prime } (0)}}{{\sqrt {{\text{Re}}_{w} } }}$$

here shear stress as

15 $$\tau_{w} = \left[ {(\mu + k_{v} )\left( {\frac{\partial u}{{\partial y}}} \right) + k_{v} N} \right]_{y = 0} = (\mu + k_{v} )\,bx\sqrt {\frac{b}{\upsilon }} f^{\prime \prime } (0)$$

and ${\text{Re}}_{w} = \frac{{u_{w} x}}{v}$: “local Reynolds number”,

The “couple stress” at the surface

16 $$M_{w} = \left( {\gamma \frac{\partial N}{{\partial y}}} \right)_{y = 0} = \mu u_{w} \left( {1 + \frac{K}{2}} \right)g^{\prime } (0)$$

The “local surface heat flux $q_{w} (x)$,the local Nusselt number $Nu_{x}$ the local mass flux $j_{w}$ and Sherwood number $Sh_{x}$” are given as follows

17 $$q_{w} (x) = - k_{f} (T_{w} - T_{\infty } )\sqrt{\frac{b}{v}} \theta^{\prime } (0)$$

18 $$Nu_{x} = \frac{xh(x)}{{k_{f} }} = - \sqrt{\frac{b}{v}} \theta^{\prime } (0) \Rightarrow \frac{{Nu_{x} }}{{\sqrt {{\text{Re}}_{w} } }} = - \theta^{\prime } (0)$$

19 $$j_{w} = - D\left( {\frac{\partial C}{{\partial y}}} \right)_{y = 0}$$

20 $$Sh_{x} = \frac{{j_{w} x}}{{D\left( {C_{w} - C_{\infty } } \right)}} = - \sqrt{\frac{b}{v}} x\phi^{\prime } (0) \Rightarrow \frac{{Sh_{x} }}{{\sqrt {{\text{Re}}_{w} } }} = - \phi^{\prime } (0)$$

## Result discussion

The essential objective of given investigation is to demonstrate the influence of several physical parameters on velocity $f^{\prime } \left( \eta \right)$, microrotation $g\left( \eta \right)$, temperature $\theta \left( \eta \right)$, and concentration $\phi \left( \eta \right)$ distributions across the available stretching sheet. Equations (9–12) together with boundary conditions (13) are evaluated numerically. Therefore, obtained results develop an excellent agreement with those retrieved by (Table 1) Tripathy et al.^19^. Later it has been determined that computed consequences had essential significant influences.

**Table 1:** Comparison of $- f^{\prime \prime } (0)$ for different values $K$ in the absence of the parameters $S = 0$, $Me = 0$, $\delta_{1} = \delta_{3} = \delta_{4} = 0$.

$K$	Tripathy et al.^26^	Present study
0.0	1.000172	1.000174
0.5	1.367902	1.367900
1.0	1.621938	1.621933

**Figure 2:**
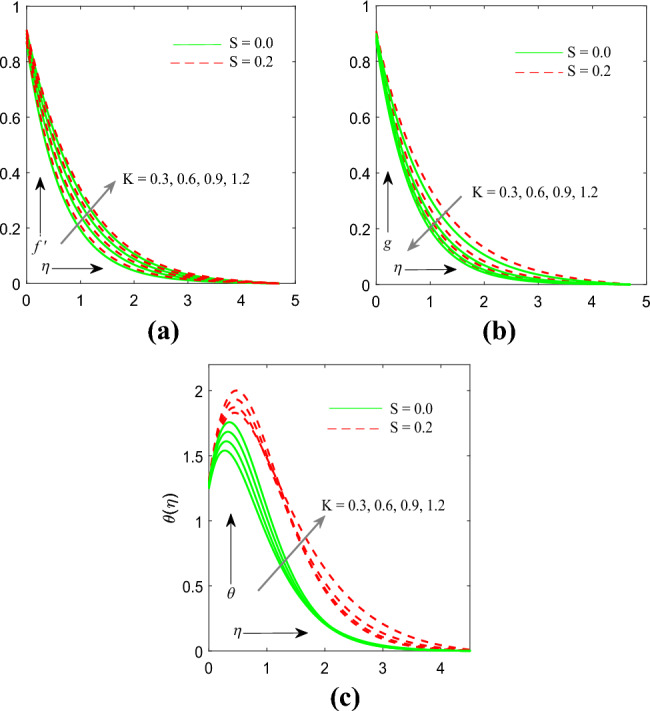
(**a**) Influence of $K$ on velocity profile. (**b**) Influence of $K$ on temperature profile. (**c**) Influence of $K$ on microrotation profile.

Figure 2a–c exemplify the consequences of material parameter $\left( K \right)$ on velocity $f^{\prime } \left( \eta \right)$, micro rotation $g\left( \eta \right)$, temperature $\theta \left( \eta \right)$ profile. Whenever values of $K$ gets increased the profiles of velocity and temperature gets enhanced but on the other hand micro rotation profile gets cut down. Physically**,** in micropolar fluids, the material parameter that can affect the velocity profile is known as the micropolar fluidity parameter (K). When the micropolar fluidity parameter (K) increases, it implies that the microstructure or internal degrees of freedom have a stronger effect on the fluid flow. This can lead to an increase in the complexity of the flow patterns and the velocity profile.

**Figure 3:**
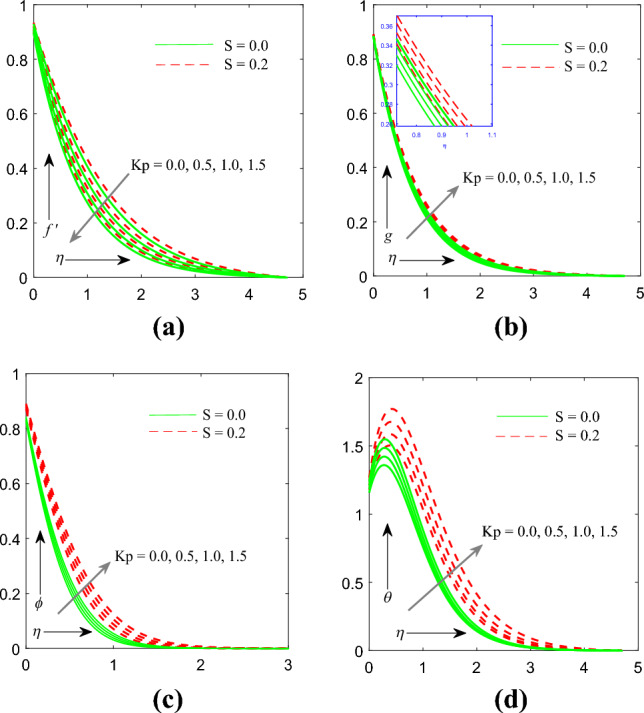
(**a**) Influence of $K_p$ on velocity profile. (**b**) Influence of $K_p$ on microrotation profile. (**c**) Influence of $K_p$ on temperature profile. (**d**) Influence of $K_p$ on concentration profile.

Figure 3a–d exhibits consequences of porosity parameter $\left( {Kp} \right)$ on velocity $f^{\prime } \left( \eta \right)$, micro rotation $g\left( \eta \right)$, temperature $\theta \left( \eta \right)$ and concentration $\phi \left( \eta \right)$ profile. Figure 3a flow stream reduces with improving values of parameter of porous medium $\left( {Kp} \right)$ or decreasing for permeability $\left( {k_{p} } \right)$. The equation of momentum reflects Darcian resistance force is inversely proportional to parameter of permeability $\left( {k_{p} } \right)$, therefore smaller permeability may lead to large Darcian resistance to the fluid flow. The field of the flow thus diminishes with increasing values of $\left( {Kp} \right)$. The profiles of micro rotation $g\left( \eta \right)$, temperature $\theta \left( \eta \right)$ and concentration $\phi \left( \eta \right)$ gets enhanced.

**Figure 4:**
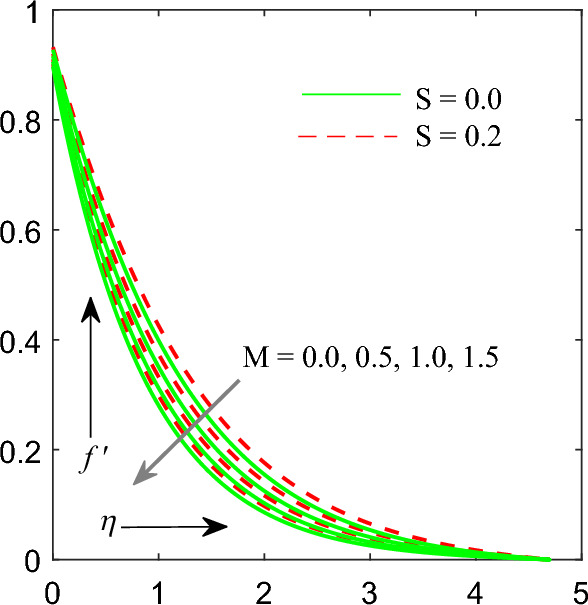
Influence of $M$ on velocity profile.

Figure 4 substantiate consequences of magnetic field parameter $\left( M \right)$ on velocity profile $f^{\prime } \left( \eta \right)$. The values of $M$ increases results decrease in the velocity profile. Lorentz force came into an existence when magnetic field imposed over flow field. This force has an intensity to drag the fluid flow by cutting down its velocity. Hence fluid flow velocity with thickness of momentum layer gets declined.

**Figure 5:**
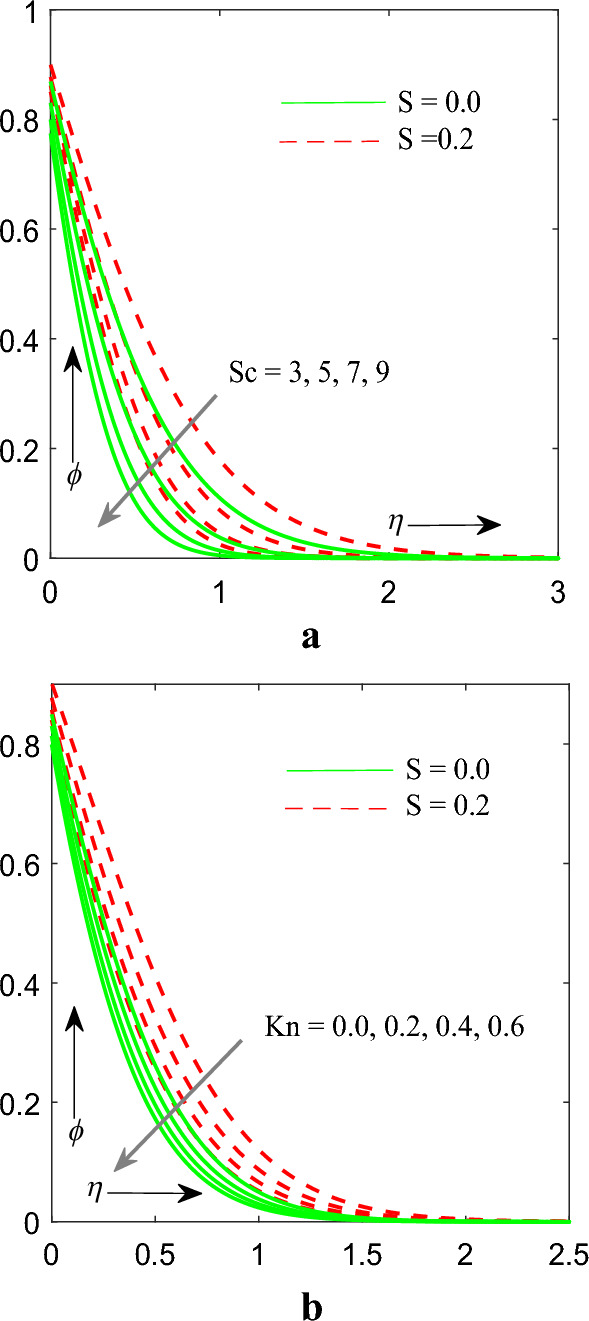
(**a**) Influence of $Sc$ on concentration profile. (**b**) Influence of $Kn$ on concentration profile.

Figure 5a,b reflects impact of Schmidt number $\left( {Sc} \right)$ and Chemical reaction $\left( {Kn} \right)$ parameter on profile of concentration $\phi \left( \eta \right)$. It has been further noticed that with the increasing value of $Sc$ and $Kn$ profile for concentration gets diminised. Physically as $Sc$ is the ratio of momentum diffusivity to mass diffusivity and when the Schmidt number increases, it means that the mass diffusivity of the fluid decreases relative to its momentum diffusivity which imply lower scalar diffusivity, resulting in reduced diffusion and slower concentration changes within the fluid medium.

**Figure 6:**
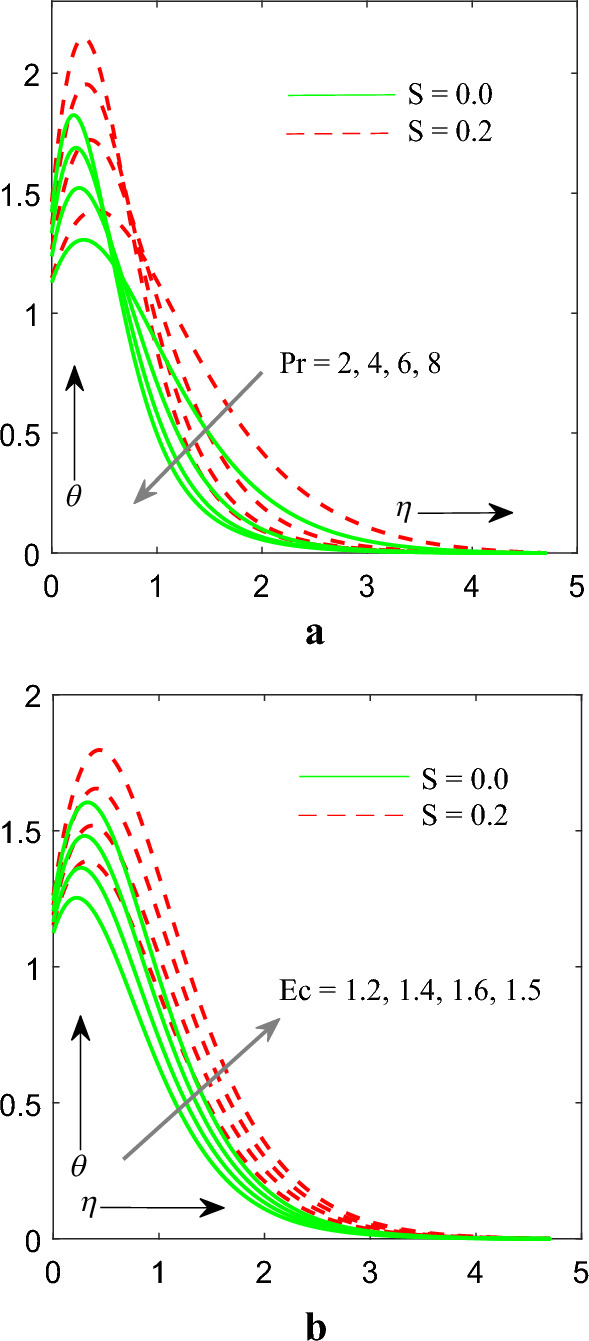
(**a**) Influence of $\Pr$ on temperaure profile. (**b**) Influence of $Ec$ on temperature profile.

Figure 6a,b demonstrate the effect of Prandtl number $\left( {\Pr } \right)$ & Eckert number $\left( {Ec} \right)$ on temperature $\theta \left( \eta \right)$ profile. We noticed that as we increase the values of $\Pr$ the temperature profile decrease, while revers effect are observed on $Ec$. Physically, it is worth mentioning that increasing values of the $Ec$ heat gets generated in the available fluid owing to application of frictional heating. Thus, improving value of $Ec$ increases the temperature within the flow of fluid.

**Figure 7:**
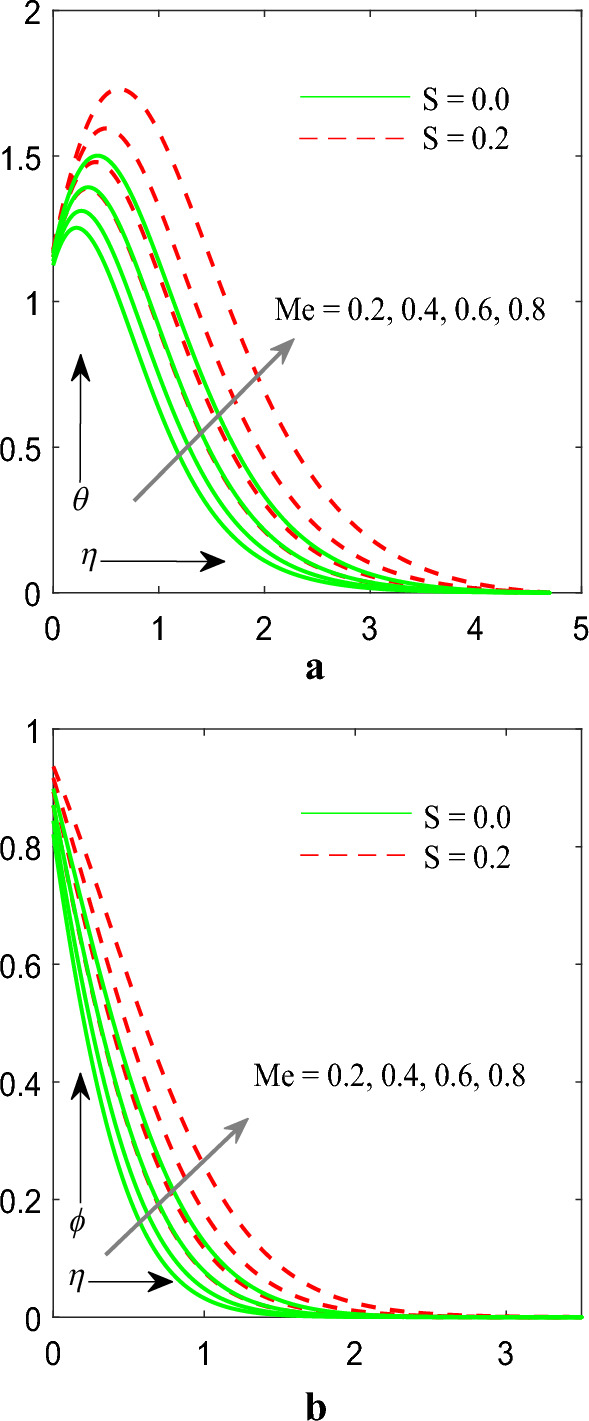
(**a**) Influence of $Me$ on temperature profile. (**b**) Influence of $Me$ on concentration profile.

**Figure 8:**
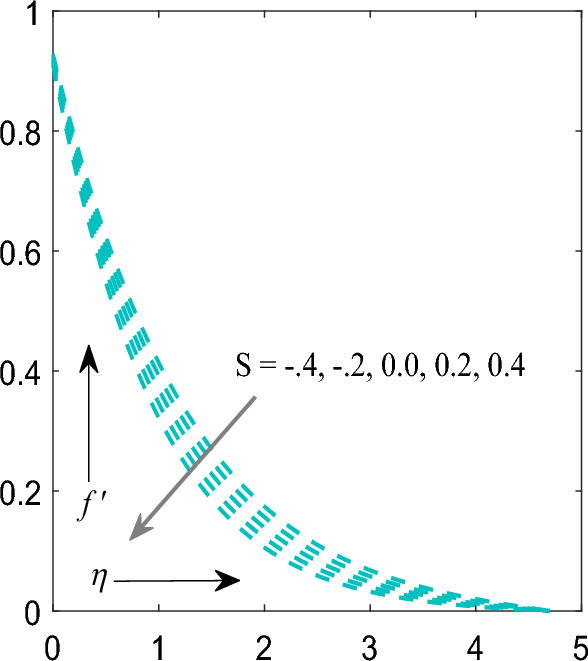
Influence of $S$ on velocity profile.

Figure 7a,b indicates effect of melting parameter $\left( {Me} \right)$ on temperature $\theta \left( \eta \right)$ as well as concentration $\phi \left( \eta \right)$ profile. It has been notified that improving values of $Me$ both profiles enhanced. Figure 8 signifies consequences of section/injection parameter $\left( S \right)$ on velocity profile $f^{\prime}\left( \eta \right)$. It is finally demonstrated that increasing values of $S$ the velocity profile decreased.

**Figure 9:**
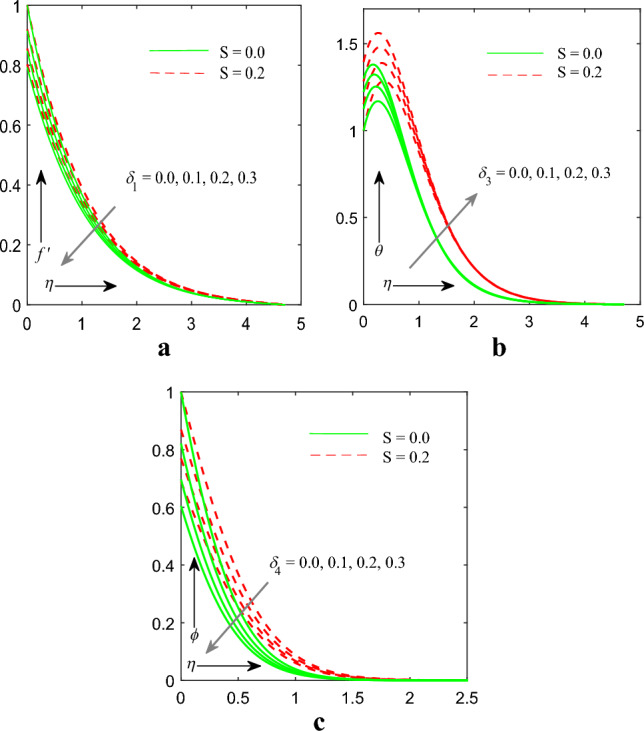
(**a**) Influence of $\delta_{1}$ on velocity profile. (**b**) Influence of $\delta_{3}$ on temperature profile. (**c**) Influence of $\delta_{4}$ on concentration profile.

Figure 9a–c reflects the effect of velocity slip $\left( {\delta_{1} } \right)$, temperature slip $\left( {\delta_{3} } \right)$ and concentration slip $\left( {\delta_{4} } \right)$ parameter on velocity $f^{\prime } \left( \eta \right)$,temperature $\theta \left( \eta \right)$ and concentration $\phi \left( \eta \right)$ profile. We observed that $f^{\prime } \left( \eta \right)$ and $\phi \left( \eta \right)$ profile get cut down on the other aspect $\theta \left( \eta \right)$ profile improved. Physically, when the slip parameter is positive, implying a positive slip velocity, the velocity profile in the fluid near the surface decreases. This is because the fluid molecules experience a relative motion along the surface, causing a reduction in their velocity close to the surface. As a result, the velocity profile exhibits a decreasing trend as you move from the surface towards the bulk of the fluid.

**Figure 10:**
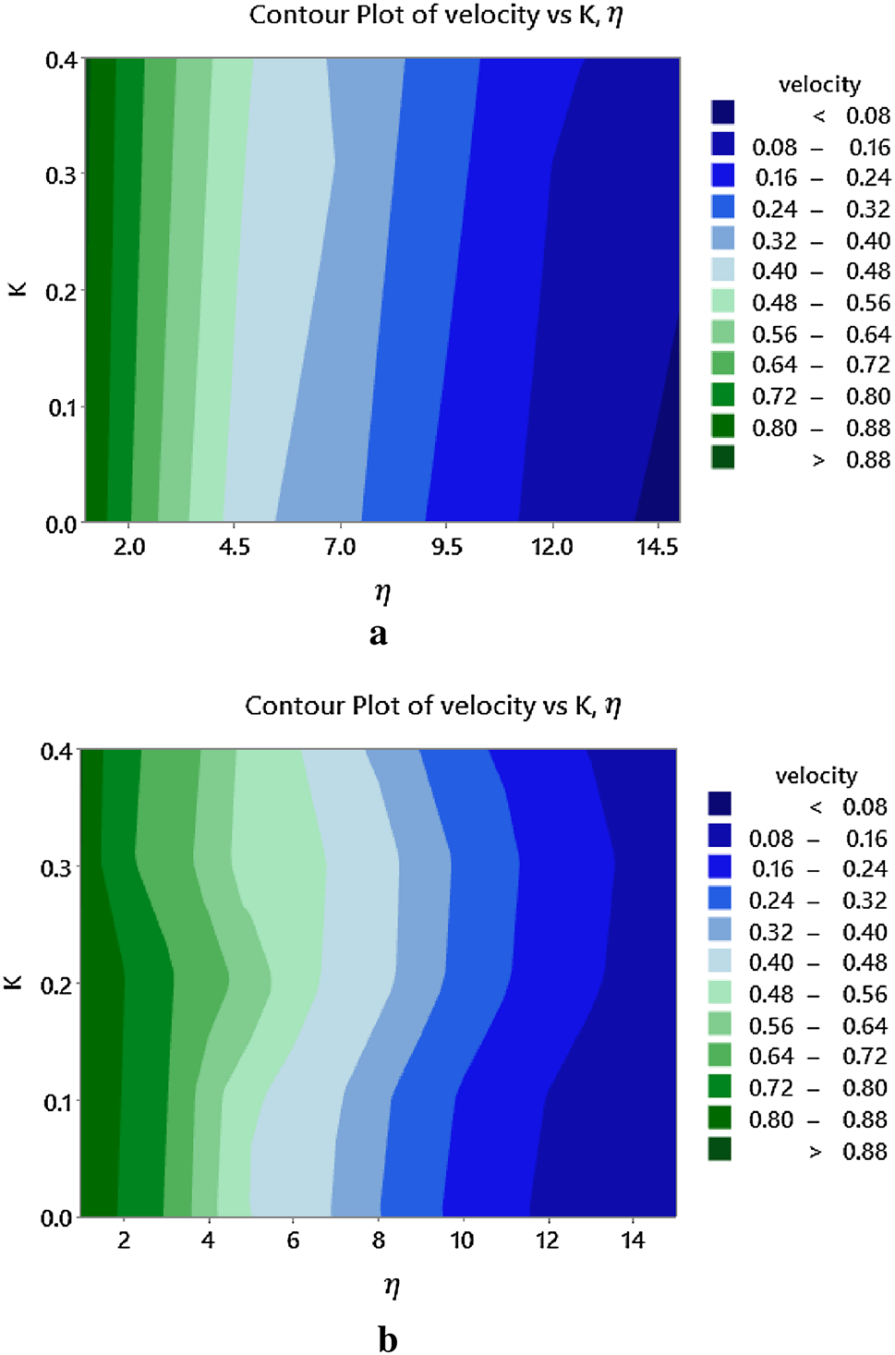
(**a**) Influence of $K$ on the velocity. (**b**) Influence of $K$ on the velocity profile when $S = 0.2$. profile when $S = 0$.

Figure 10a,b demonstrates the change in the velocity profile with respect to the increasing micro-rotation parameter, $K$, for two cases, such as: (i) $S = 0.0$ and (ii) $S = 0.2$. For both the cases, it is observed that the velocity is more intense in the region close to the surface than in the ambient regions. Near the surface, the surface effects that arise from various phenomena such as intermolecular forces, surface tension, or boundary layer interactions can become more dominant and can affect the behavior of the micropolar fluid more strongly. Further, the increased micro-rotation parameter amplifies the influence of the rotational motion near the surface, leading to a more intense impact on the fluid velocity.

**Figure 11:**
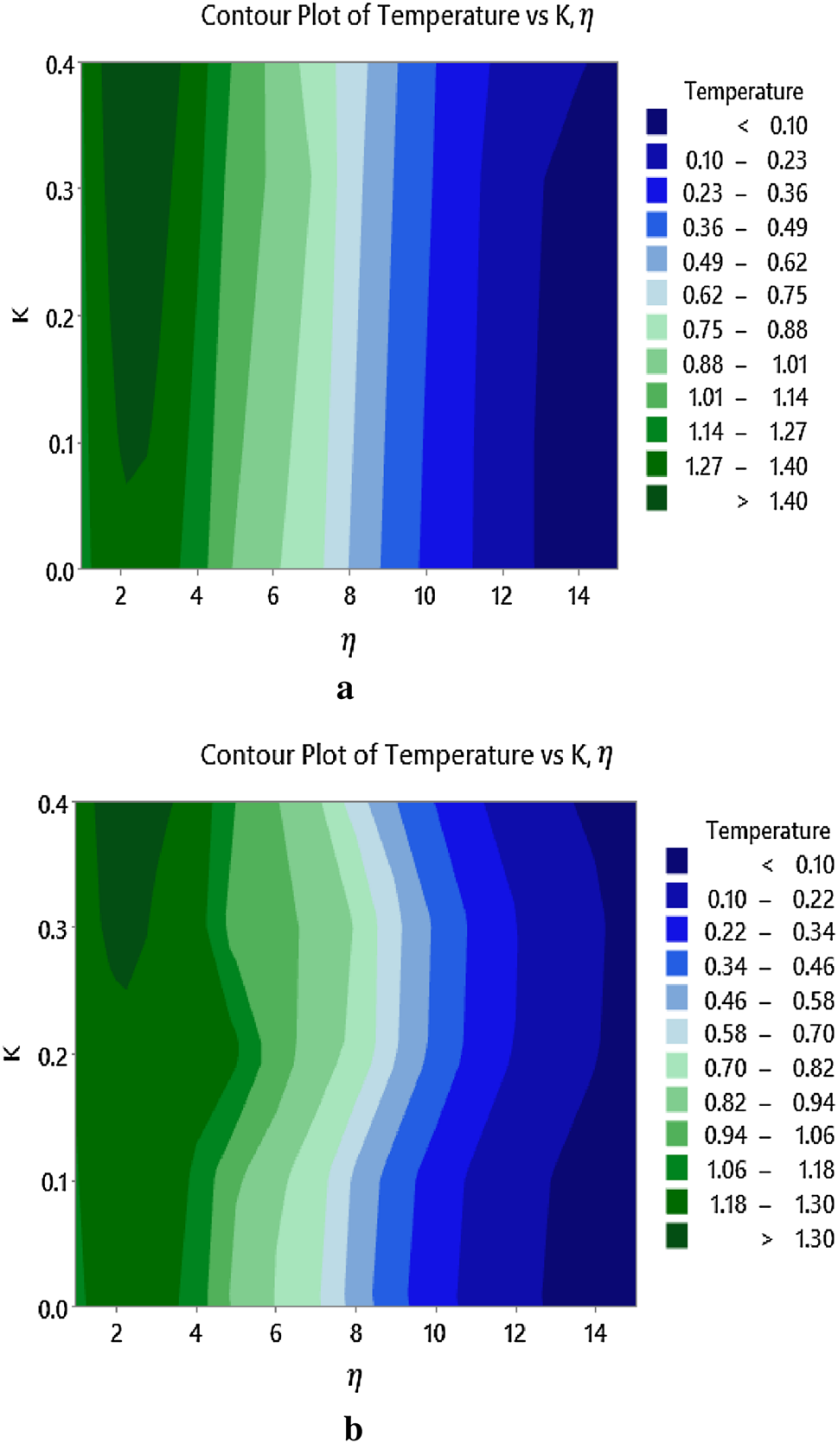
(**a**) Influence of $K$ on the temperature profile when $S = 0.2$. (**b**) Influence of $K$ on the temperature. profile when $S = 0$.

Contours showing the impact of micro-rotation parameter, $K$, on the temperature is shown in Fig. 11a for the case when $S = 0.0$ and Fig. 11b when $S = 0.2$, respectively. It is obvious from the figures that the temperature increases with increasing $K$. Physically, the micro-rotation parameter affects the rotational motion of fluid elements, which can impact the flow patterns near the surface and alter the convective heat transfer processes. The altered flow patterns, in turn, can influence the heat transfer mechanisms and distribution of temperature near the surface.

Moreover, the micro-rotation parameter affecting the velocity and temperature of a micropolar fluid more intensely near the surface slightly varies according to the boundary conditions (i.e. when $S = 0.0$ and $S = 0.2$), which is clearly visible via Figs. 10a and 11b. From this, in general, it is concludable that the micro-rotation parameter's effect on the velocity and temperature of a micropolar fluid is typically influenced by factors such as suction and injection.

## Conclusions

In the present analysis, a numerical investigation of micro polar fluid flow due to melting stretchy surface in a porous medium has been carried out. The influence of abundant quantities on velocity, microrotation, temperature and concentration distribution are outlined as follows:

- The velocity $f^{\prime } \left( \eta \right)$ and temperature $\theta \left( \eta \right)$ profile it noticed rising with increasing amount of $K$ however, micro-ration profile $g\left( \eta \right)$ gets cut down.
- The influence of $K_p$ is observed to enhance $\theta \left( \eta \right)$ profile however velocity $f^{\prime } \left( \eta \right)$ gets cut down.
- The concentration profile $\phi \left( \eta \right)$ decrease with increasing values of the parameters $Sc$ and $Kn$.
- Reduction in velocity $f^{\prime } \left( \eta \right)$ profile is manifested with an increase in value of slip parameters $\left( {\delta_{1} } \right)$.

## Data Availability

Data analysed during this study are included in this published article.

## List of symbols

$B_{0}$: Strength of magnetic field (kg s^−^^2^ A^−^^1^)
$C$: Fluid’s concentration (kg m^−^^3^)
$C_{f}$: Skin friction coefficient
$C_{p}$: Specific heat (J kg^−^^1^ k^−^^1^)
$$c_{s}$$: Heat capacity (J K^−^^1^)
$C_{w}$: Fluid concentration at the wall (kg m^−^^3^)
$D$: Coefficient of mass diffusion (m^2 ^s^−^^1^)
$${\text{Ec}}$$: Eckert number
$K$: Material (micropolar) fluid parameter
$$k_{f}$$: Thermal conductivity (W/m K)
$k_{v}$: Vortex viscosity (N sm^2^)
$K_{n}$: Chemical reaction parameter
$$K_{p}$$: Porous parameter
$L_{1}$: Velocity slip factor
$L_{2}$: Thermal slip factor
$L_{3}$: Concentration slip factor
$M$: Magnetic field parameter
$Me$: Melting surface parameter
$N$: Microrotation/angular velocity (s^−^^1^)
${\text{Nu}}_{x}$: Local Nusselt number
$$\Pr$$: Prandtl number
$q_{w} (x)$: Local surface heat flux (W m^−^^2^)
${\text{Re}}_{w}$: Local Reynolds number
$$S$$: Suction/injection
$${\text{Sc}}$$: Schmidt number
$${\text{Sh}}_{x}$$: Sherwood number
$T_{0}$: Solid surface temperature (K)
$$T_{m}$$: Melting temperature (K)
$T_{\infty }$: Free stream temperature (K)
$$u_{w}$$: Surface velocity (m s^−^^1^)
$u,\,v$: Velocity component corresponding to horizontal and the vertical direction (m s^−^^1^)
$\alpha ,\,\beta$: Source dependent and Temperature dependent parameter
$\rho$: Fluid density (kg m^−^^1^)
$$\upsilon$$: Kinematic viscosity (m^2 ^s^−^^1^)
$$\sigma$$: Electrical conductivity (s m^−^^1^)
$\beta_{m}$: Latent heat (J kg^−^^1^)
$$\gamma$$: Reference length (m)
$\delta_{1}$: Velocity slip parameter
$\delta_{2}$: Temperature slip parameter
$\delta_{3}$: Concentration slip parameter
$f^{\prime } \left( \eta \right)$: Non-dimensional velocity parameter
$g\left( \eta \right)$: Non-dimensional microrotation parameter
$\theta \left( \eta \right)$: Non-dimensional temperature parameter
$\phi \left( \eta \right)$: Non-dimensional concentration parameter
